# Adherence to the Mediterranean Diet and Risk of Depression: A Cohort Study in Chinese Community Residents

**DOI:** 10.3390/nu17060942

**Published:** 2025-03-07

**Authors:** Kexin Zhang, Yanan Wu, Liping Yi, Yiling Wu, Yingqi Deng, Xinxin Xu, Biying Wang, Yonggen Jiang, Qi Zhao, Genming Zhao

**Affiliations:** 1 Key Laboratory of Public Health Safety of Ministry of Education, Department of Epidemiology, School of Public Health, Fudan University, Shanghai 200032, China; 2 Songjiang District Center for Disease Control and Prevention, Shanghai 201600, China

**Keywords:** depression, dietary pattern, Mediterranean diet, aMED, cohort study

## Abstract

**Objectives:** Limited studies have investigated the association between compliance with a Mediterranean diet and depression in China. We sought to explore the potential association between the adherence to an alternate Mediterranean diet score (aMED) and the risk of developing depression among adults in Eastern China. **Methods**: This study used a prospective cohort design that involved a total of 52,232 individuals in the Shanghai Suburban Adult Cohort and Biobank (SSACB). A reliable food frequency questionnaire (FFQ) was utilized to evaluate dietary intake, and we calculated the aMED score for each participant, dividing them into 3 groups accordingly (score 0–3, 4–5, 6–9). Cox proportional hazards regression analyses were performed to compute the hazard ratios (HR) and 95% confidence intervals (CI). **Results**: The median age of participants was 58 years (IQR: 50–65), with a male-to-female ratio of 1:1.59. Throughout an average 6.29 years of follow up, 1220 incident cases of depression were recorded through the ICD-10 classification codes F32 and F33. A higher level of adherence to the aMED was notably linked to a decreased risk of incident depression (HR_high vs. low adherence_ = 0.83, 95% CI = 0.70–0.98; HR_moderate vs. low adherence_ = 0.87, 95% CI = 0.76–0.99; *P*-trend = 0.009) after multivariate adjustment. Each 1-score increase in the aMED score was associated with a 5% lower risk of depression (HR = 0.95, 95% CI = 0.91–0.99), and this association was more pronounced among participants aged ≥65 years (*P*-interaction = 0.008). **Conclusions**: Our results suggest that following a Mediterranean diet might potentially provide mental health benefits, particularly for individuals aged 65 years and above.

## 1. Introduction

As a prevalent mental health disorder, depression affects an estimated 320 million people globally, with its incidence rapidly increasing in recent years. During the COVID-19 pandemic, depression further aggravated the global disease burden [1]. The World Health Organization (WHO) forecasts that depression will emerge as a primary global contributor to the disease burden by 2030 [2], underscoring its profound impact on individuals and societies. In China, depression affects approximately 54 million individuals, with lifetime and 12-month prevalence rates estimated to be 6.8% and 3.6%, respectively [3]. These figures reflect a growing public health challenge in China and highlight the urgent need for research and interventions targeting depression.

To date, numerous studies have explored the potential risk factors associated with depression from both clinical and public health perspectives [4,5]. However, modifiable risk factors for depression are not yet fully clarified. Current evidence suggests that several modifiable factors may increase the risk of depression, including lifestyle factors such as sleep disorders [6], lack of physical activity [7], and unhealthy diet [8], as well as psychosocial aspects like chronic environmental stress [9] and social isolation [10]. Recently, diet has emerged as a promising modifiable target for preventing depression [11,12,13,14]. Instead of focusing solely on particular nutrients or specific foods, analyzing overall dietary patterns provides a broader understanding of diet’s influence on health outcomes [15]. Among dietary patterns, the Mediterranean diet (MD) stands out for its potential role in supporting mental and brain health [16,17,18]. The alternate Mediterranean diet score (aMED), derived from Trichopoulou and colleagues’ Mediterranean diet scale [19,20], highlights greater consumption of plant-derived foods (e.g., vegetables, legumes, and fruits) and monounsaturated fats, along with limiting saturated fats and processed meats, and allowing moderate alcohol intake. While maintaining the core features of the traditional MD, the aMED has been recommended in diverse populations beyond the Mediterranean region [21,22,23].

Increasing evidence indicates that adherence to the MD is associated with improved mental health outcomes, including enhanced attention, greater contentment, and reduced anxiety symptoms [24,25]. However, despite these broad mental health benefits, the association between MD and depression remains inconsistent. Observational studies have yielded mixed findings regarding the impact of MD adherence on depression [13,26,27]. The pooled result of a meta-analysis including nine cross-sectional studies revealed a notable negative correlation between following the MD and depression risk [26]. In contrast, evidence from cohort studies remains inconsistent [12,28,29,30,31], possibly because of differences in demographic features, follow-up duration, and confounding variables [12,28]. More importantly, the clinical definitions and diagnostic criteria for depression differ across these studies.

While numerous studies have focused on Western populations, research on the MD and its impact on mental health in Chinese populations remains limited. Most existing studies in China have primarily investigated specific foods or nutrients, such as vegetables or omega-3 fatty acids [32,33,34], with little attention to Mediterranean-style dietary patterns. Given the different dietary habits of China and Western countries, whether findings from Western cohorts are fully applicable to Chinese contexts remains uncertain. Although the aMED was formulated to ensure broader applicability, few studies in China have yet examined aMED adherence in relation to depression risk. Therefore, well-designed research is essential to confirm the conclusions of previous studies among Chinese populations.

Thus, the aim of this study was to investigate the prospective association between adherence to the aMED and the risk of developing depression in a community-based cohort in Eastern China.

## 2. Materials and Methods

### 2.1. Subjects and Source

Data were obtained from the Shanghai Suburban Adult Cohort and Biobank (SSACB), a prospective cohort established between 2016 and 2019 in China. Details of this cohort have been reported in our prior publication [35]. In brief, a multi-stage, stratified, and randomized cluster sampling approach was employed at the beginning to ensure the representativeness of the cohort. Initially, twelve communities were chosen from four districts of Shanghai—Songjiang, Jiading, Minhang, and Xuhui—based on several factors, including demographic scale, location characteristics, and accessibility to healthcare services. Following this, one-third of the neighborhoods or villages within each community were randomly selected to participate. Qualified subjects were adults aged 20–74 years who had resided in these selected neighborhoods or villages for at least five years prior to the study. Residents were formally recruited into the cohort after obtaining their informed consent.

Between June 2016 and October 2019, 69,116 participants were recruited to the SSACB in total. Baseline data were gathered using face-to-face interviews, which were carried out by qualified personnel employing structured questionnaires. In addition, physical examinations and biochemical tests were also conducted at baseline. Each subject was allocated a personalized identification number to be linked to the Electronic Medical Record System (EMRS) and the Cause-of-Death Surveillance System (CDSS) of Shanghai. These databases provided longitudinal data on participants’ health outcomes, with disease diagnoses classified according to the International Classification of Diseases, Tenth Revision (ICD-10).

**Figure 1:**
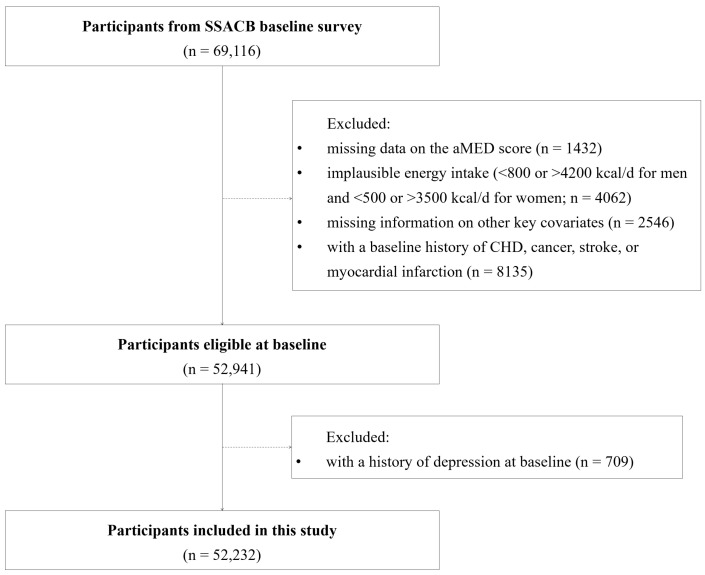
Flowchart of the study participants’ recruitment.

We excluded participants who had incomplete intake data of food items related to the aMED score, implausible energy intake (<800 or >4200 kcal per day for men, and <500 or >3500 kcal per day for women), or missing information on other key covariates, including physical activity and body mass index (BMI). Additionally, individuals with baseline history of CHD, cancer, stroke, or myocardial infarction were removed. Among the remaining participants, those with a baseline history of depression were also excluded. Finally, 52,232 participants were enrolled in this study, with a final response rate of 75.6% (Figure 1).

### 2.2. Dietary Assessment

A self-reported food frequency questionnaire (FFQ) was designed to collect information on the average food intake of participants during the previous year. The FFQ included 29 groups of foods, considering the traditional dietary habits of Shanghai residents. For each food group, participants reported both the frequency of consumption (categorized as: never, less than once/month, 1–3 times/month, 1–3 times/week, 4–6 times/week, once/day, twice/day, or more than 3 times/day) and the typical portion size consumed per occasion (measured in grams or milliliters) over the preceding 12 months. These values were then multiplied together to estimate the daily average intake of each food item. The daily intake of legumes was determined by summing the servings of tofu (60 g/serving) and soy milk (150 g/serving) consumed per day in this study. Nutrients and energy from each food item were calculated based on the Chinese Food Composition Table [36].

The aMED score was derived from the Mediterranean diet scale created by Trichopoulou and colleagues [19,20], and comprised nine dietary components: vegetables (excluding potatoes), fruits, nuts, whole grains, legumes, fish, the ratio of monounsaturated to saturated fats, red and processed meats, and alcohol. For dietary components considered beneficial—namely, vegetables, fruits, nuts, whole grains, legumes, fish, and the ratio of monounsaturated to saturated fats—participants received a score of 1 if their intake met or exceeded the population’s gender-specific median level. Otherwise, they received 0 for these food components. For red and processed meats, on the other hand, participants whose intake levels below the gender-specific median were assigned 1, while those at or above the median were given 0. Regarding alcohol intake, women with a consumption of 5–15 g/day and men of 10–25 g/day were assigned a score of 1, while all other levels of consumption were assigned 0. The scores for these nine ingredients were summed for each individual to calculate an aMED score, with a range between 0 and 9, where increased values reflected closer concordance to the Mediterranean diet.

We initially analyzed the aMED score as a categorical variable by dividing it into predefined categories of low (0–3), moderate (4–5), and high (6–9), with the low category being the reference group, as previously described in related studies [22,37,38]. Subsequently, to estimate the association between per unit increase in aMED score and the risk of depression, we analyzed the score as a continuous variable in regression models.

### 2.3. Diagnosis of Depression

The primary outcome assessed in this study was the earliest documented clinical diagnosis of depression during the follow-up period. Incident depression cases within the SSACB were identified using data from the EMRS of Shanghai, which encompasses both outpatient and inpatient medical records. Diagnoses were coded using the International Classification of Diseases, 10th Revision (ICD-10). Depression cases were defined as those with ICD-10 codes F32 and F33 [39]. To ensure up-to-date case identification, hospital records and death registries were included up to 31 March 2024.

### 2.4. Assessment of Covariates

Baseline data on potential confounding variables were collected through self-reported questionnaires, physical examinations, and biochemical tests. Demographic characteristics included age, gender, smoking status, marital status, educational attainment, and retirement status. Anthropometric measurements, including weight and height, were obtained during physical examinations, and BMI was determined by dividing the weight (in kilograms) by the square of the height (in meters). Lifestyle factors were also assessed. Physical activity was evaluated using the International Physical Activity Questionnaire (IPAQ) guidelines [40], with results expressed as metabolic equivalent of task (MET) hours per week. Sleep quality was quantified using the Pittsburgh Sleep Quality Index (PSQI) [41], which provides a composite score based on self-reported sleep patterns. Dietary energy intake was evaluated from the FFQ, incorporating the Chinese Food Composition Table. Clinical histories of hypertension, diabetes, hyperlipidemia, dementia, and Parkinson’s disease were ascertained through both self-reports and verified medical records. Family history of depression was also recorded at baseline.

### 2.5. Statistical Analysis

The duration of follow-up for each participant in the SSACB was measured in person-years, starting from the baseline interview and continuing until the occurrence of one of the following events: incident depression, death, or the end of this study (31 March 2024). We applied the Kolmogorov–Smirnov test to assess the normality of continuous variables. Medians (P25, P75) and percentages were used to describe the continuous and categorical variables, respectively. To compare differences between aMED score groups, the Kruskal–Wallis rank-sum test was applied for continuous variables, while the Mantel–Haenszel χ^2^ test was used for categorical variables.

Multiple Cox proportional hazard regression models were applied to calculate hazard ratios (HRs) and 95% confidence intervals (CIs) for the risk of incident depression across aMED adherence groups. Model 1 was only adjusted for age (continuous variable, years) and gender (male, female). Model 2 was further adjusted for BMI (normal [<24.0], overweight [24.0–28.0], and obese [>28.0 kg/m^2^]), smoking status (never, quit ≥ 10 years, quit < 10 years, current), educational attainment (junior high school or below, high school or above), total energy intake (quintiles, kcal/day), physical activity (tertiles, MET-h/week), marital status (married, unmarried, divorced or other), retirement status (yes, no), PSQI (continuous variable), and family history of depression (yes, no). Model 3, the fully adjusted model, was additionally adjusted for individual history of hypertension, diabetes, hyperlipidemia, dementia, and Parkinson’s disease (each as yes or no). Schoenfeld residuals were used to test the proportional hazards assumption, and no evidence of violation was detected in our analyses. The *p*-value for the linear trend was calculated by analyzing the aMED score as a continuous variable. To explore potential nonlinear correlation between the aMED score and depression risk, a restricted cubic spline (RCS) model with three knots (corresponding to the 10th, 50th, and 90th percentiles) was employed.

Potential effect modifications of socio-demographic factors and history of chronic diseases on the aMED score were evaluated by introducing interaction terms in Model 3. Stratified analyses were also carried out within the same adjusted model, where participants were grouped by age (<65, ≥65 years), gender (male, female), BMI level (normal, overweight, obese), educational attainment (junior high school or below, high school or above), retirement status (yes, no), marital status (married, unmarried or other), current smoking status (yes, no), physical activity level (tertiles, MET-h/week), PSQI level (tertiles), and history of diseases (hypertension, diabetes, and hyperlipidemia, each yes or no). Each subgroup analysis was adjusted for all confounding variables included in Model 3 except for the stratified variable itself.

To validate the consistency and dependability of our findings, we conducted several sensitivity analyses. Firstly, participants who were diagnosed with depression during the initial one and two years of follow-up, respectively, were excluded from the cohort, to avoid possible reverse causation. Secondly, given the controversial impacts of alcohol consumption on health outcomes [42,43], alcohol was removed from the components of the aMED score. The scores of the remaining eight food components were subsequently aggregated to calculate the modified aMED score, and participants were categorized by their concordance: low (0–2), moderate (3–5), and high (6–8). However, in regression models, we made additional adjustments for drinking status (never, stopped ≥ 10 years, stopped < 10 years, current). Furthermore, we performed a data-driven classification method to divide the aMED score of all participants into quartiles, with quartile 1 (Q1) as the reference group.

A *p* value of less than 0.05 was deemed to signify a statistically significant difference. R V.4.3.2 software was used for all statistical analyses.

## 3. Results

### 3.1. Baseline Characteristics and the Incidence of Depression

This study included 52,232 participants, with 61.3% (*n* = 32,031) being female. At baseline, the median age of participants was 58 years (IQR: 50–65), as shown in Table 1. Participants who had higher adherence to the aMED tended to be male, with better educational attainment, and had higher dietary energy intake compared to those with lower adherence. Detailed dietary intake of aMED components across adherence levels are presented in Table S1.

During the follow-up period from 2016 to 2024 (mean ± SD = 6.29 ± 1.26 years), 1220 cases of incident depression were identified, with an incidence density of 3.72/1000 person-years (95% CI: 3.51–3.93) and a cumulative incidence of 2.34% (95% CI: 2.21–2.47). Stratified by age, the incidence density and cumulative incidence for participants aged 65 years and above were 4.46/1000 person-years (95% CI: 4.02–4.95) and 2.64% (95% CI: 2.37–2.92), respectively. In contrast, for participants younger than 65 years, the incidence density was 3.48/1000 person-years (95% CI: 3.25–3.72), with a cumulative incidence of 2.23% (95% CI: 2.08–2.38), indicating a slightly higher rate of depression incidence in the older population.

### 3.2. Association Between the aMED and Depression

**Figure 2:**
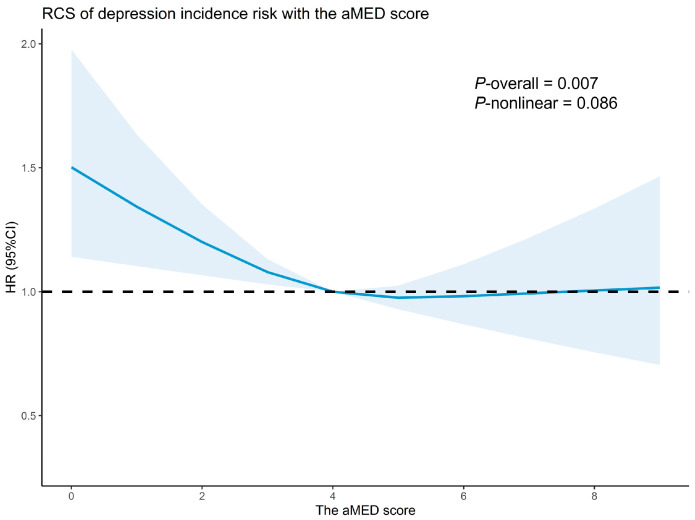
Restricted cubic spline (RCS) curve of depression risk with aMED score. The model was adjusted for age, gender, educational attainment, BMI, marital status, retirement status, total energy intake, physical activity, PSQI, smoking status, family history of depression, and history of hypertension, diabetes, hyperlipidemia, dementia, and Parkinson’s disease (the same as the covariates in Model 3). The blue continuous line indicates the hazard ratio (HR), while the shaded area represents the 95% confidence interval (CI). The black dashed horizontal line denotes the reference line (HR = 1).

Compared with participants who had low adherence to aMED (0–3), moderate (4–5) and high (6–9) adherence were associated with statistically significant reductions in the risk of incident depression (*p* < 0.05). Specifically, participants in the moderate adherence group had a 13% lower risk of depression (HR = 0.87, 95% CI = 0.76–0.99), while those in high adherence group experienced a 17% reduced risk (HR = 0.83, 95% CI = 0.70–0.98), illustrating a potential trend of decreasing depression risk with higher adherence levels (*p*-trend = 0.009) (Table 2). Furthermore, for per unit increase in the aMED score, there was an associated 5% lower risk of depression (HR = 0.95, 95% CI = 0.91–0.99). The restricted cubic spline (RCS) analysis further confirmed these findings, showing a monotonic, linear decline in depression risk as the aMED score increased (Figure 2). No significant nonlinear association was observed between the aMED score and depression (*p*-nonlinear  >  0.05).

### 3.3. Subgroup Analyses

**Figure 3:**
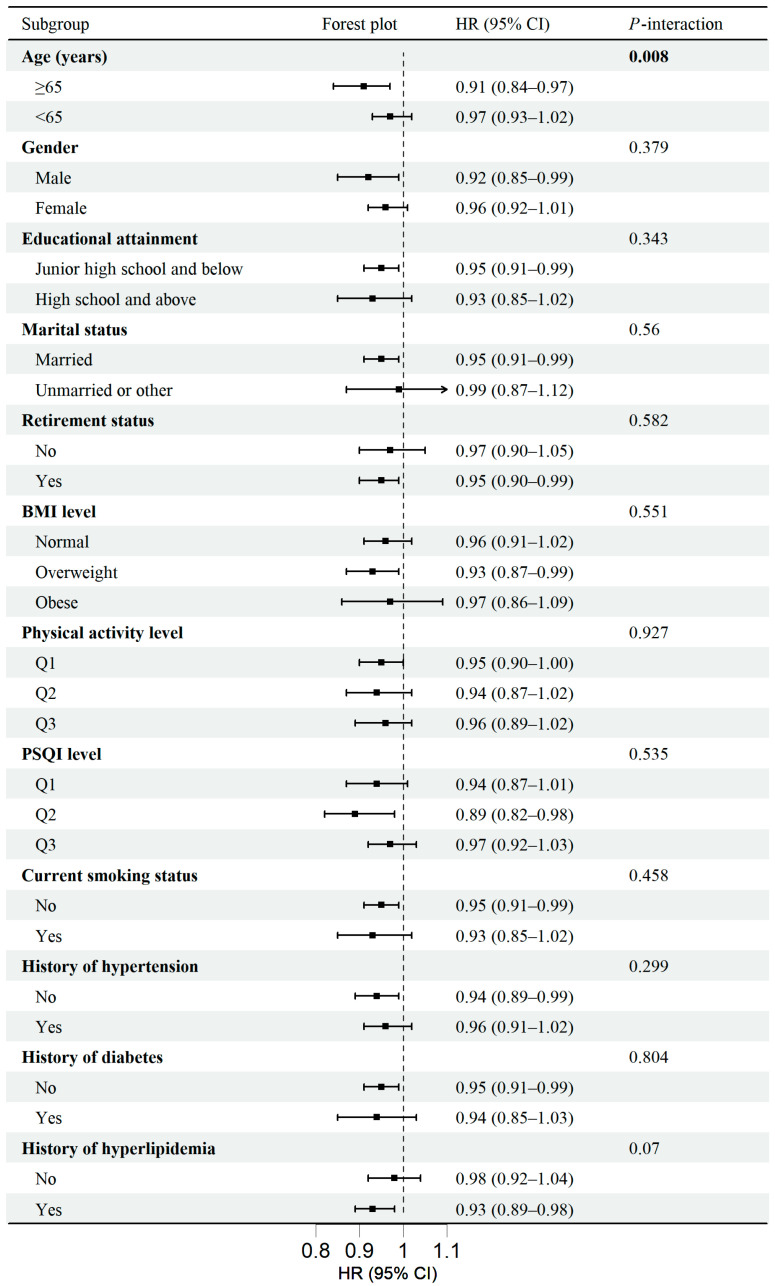
Stratified analyses of per unit increase in aMED score with risk of depression. The model was adjusted for age, gender, educational attainment, BMI, marital status, retirement status, total energy intake, physical activity, PSQI, smoking status, family history of depression, and history of hypertension, diabetes, hyperlipidemia, dementia, and Parkinson’s disease (the same as the covariates in Model 3). Each subgroup analysis was adjusted for all the covariates listed above except for the stratified variable itself. Arrows are used where the upper confidence intervals exceed 1.1.

We conducted stratified analyses to examine potential interactions between per unit increase in the aMED score and key demographic or lifestyle factors in relation to depression risk. Notably, a significant effect modification between the aMED score and age group was detected (*p*-interaction = 0.008). Specifically, the association was more pronounced among participants aged ≥65 years (HR = 0.91, 95% CI = 0.84–0.97, per unit increase) compared with those aged <65 (HR = 0.97, 95% CI = 0.93–1.02, per unit increase) (Figure 3), suggesting a stronger protective effect of aMED adherence in older adults. Additionally, while the interaction between history of hyperlipidemia and the aMED score was not statistically significant (*p*-interaction = 0.07), the subgroup HRs suggested a potential differential effect, with an enhanced association among participants with hyperlipidemia history (HR = 0.93, 95% CI = 0.89–0.98) compared to those without hyperlipidemia history (HR = 0.98, 95% CI = 0.92–1.04).

We also assessed the relationships between the aMED score and depression across other subgroups stratified by gender, BMI, PSQI, physical activity, educational attainment, smoking status, marital status, retirement status, and history of hypertension and diabetes. Across these subgroups, the correlations showed overall consistency. No statistically significant interactions were detected for any of these covariates.

### 3.4. Sensitivity Analyses

Several sensitivity analyses were performed to evaluate the robustness of our findings. Firstly, we conducted lag-time analyses by excluding participants who were diagnosed with depression within the initial one and two years of follow-up. As a result, the findings remained robust and significant. Specifically, the HRs (95% CI) for the highest adherence to aMED upon the removal of one and two years were 0.81 (0.66, 0.98) and 0.75 (0.59, 0.95), respectively (Table S2). Given the controversial impact of alcohol consumption on health outcomes [42,43], another sensitivity analysis was performed by removing alcohol from the aMED components. The result remained consistent with that of the original aMED with complete composition (HR_moderate versus low_ = 0.80, 95% CI = 0.70–0.93; HR_high versus low_ = 0.76, 95% CI = 0.62–0.93) (Table S3). Additionally, we used a data-driven approach to divide all participants into four groups based on the quartiles of the aMED score, and found that the group with higher aMED scores still experienced a significantly lower likelihood of developing depression than the one with lower scores (HR_Q2 versus Q1_ = 0.855, 95% CI = 0.735–0.995; HR_Q4 versus Q1_ = 0.830, 95% CI = 0.697–0.988) (Table S4), in accordance with the results in our main analysis.

## 4. Discussion

In this large-scale prospective cohort study, we observed that, compared to lower adherence, higher adherence to the alternate Mediterranean diet score (aMED) was linked to a reduced risk of incident depression, particularly among individuals who were 65 years of age or older.

Our results align with those reported in several earlier studies, although many were constrained by differences in exposure measures, outcome definitions, and research designs. A cross-sectional study performed among the elderly (65 years old and above) living in Mediterranean islands noted an inverse correlation between high compliance to the Mediterranean diet (MD) and a decreased risk of depression symptoms (OR = 0.65, 95% CI = 0.50–0.85) [44]. In our study, we observed similar associations in those aged 65 and above, but not in younger populations. This highlights the necessity for further studies to identify the age group that would benefit most from dietary interventions, allowing for targeted prevention of depression. Another cross-sectional study of Korean adults observed that higher modified alternate Mediterranean diet scores (mMED) were correlated with a 42% decreased likelihood of depression (OR = 0.58, 95% CI = 0.37–0.90) [45]. Furthermore, a meta-analysis of four longitudinal studies indicated a protective effect of higher MD adherence against depressive outcomes (HR = 0.67, 95% CI = 0.55–0.82) [26]. Additionally, a meta-analysis of five randomized controlled trials found that MD interventions notably alleviated depressive symptoms among middle-aged adults suffering from major depression compared to control groups [46]. Taken together, these studies, including ours, have emphasized the preventive effect of the MD against depression. Our findings also provided further evidence regarding age-related variations in this beneficial relationship.

Several theories have been suggested to explain how the Mediterranean diet might influence the diminished risk of depression. Specially, the MD is rich in bioactive compounds such as carotenoids and polyphenols that are found in plant-based food, omega-3 fatty acids that are crucial for the well-being of the brain, essential vitamins, and dietary fiber primarily derived from fruits and vegetables. Additionally, key trace elements like selenium and zinc may contribute to the overall health benefits [47,48]. These nutrients, recognized for their anti-inflammatory and antioxidant effects, could influence the key biological processes related to depression, including oxidative stress and inflammation [8,49]. Other potential mechanisms may be positively influenced by adherence to the MD. The MD was found to improve gut microbiota diversity and bacterial abundance, thereby influencing neuropsychiatric disorders via the microbiota–gut–brain axis [50,51,52]. Additionally, the impairment in neuroprotective functions afforded by brain-derived neurotrophic factor (BDNF) may mediate progressive neuronal dysfunction [53]. MD intervention, however, has been found in a randomized clinical trial to have an association with a higher plasma BDNF level in individuals with depression [54]. Moreover, MD was suggested to improve the HDL triglyceride metabolism [55], which is associated with the future risk of depression [56]. Nevertheless, further studies focusing on biomarkers related to these pathways are needed to fully explore and identify the complex underlying mechanisms [8].

The protective impact of adherence to the MD against incident depression indicated significant effect-modification by age group in our study. In particular, the correlation between the aMED score and depression was stronger among participants aged 65 years or older than in those under 65 years of age. One possible explanation is that older adults may be more susceptible to depression than younger individuals. In our study, the incidence of depression was higher among those aged 65 and older compared to younger participants, which is consistent with recent data from the Chinese Center for Disease Control and Prevention (CDC) [57]. Moreover, this age-specific difference may be partly due to physiological changes associated with aging. It is well established that inflammation and oxidative stress tend to increase with advancing age [58], both of which are considered to be key factors in the pathophysiology of depression [59,60]. The MD, recognized for its antioxidant and anti-inflammatory properties [61,62], may thus provide greater health benefits to the elderly population by targeting these age-related biological processes. In addition to age, we also found a stronger beneficial effect of the MD in participants with a baseline history of hyperlipidemia, although it was not statistically significant. This effect could be explained by the ability of MD to improve lipid profiles and reduce inflammation [52,63], given that hyperlipidemia is often accompanied by increased systemic inflammation [64,65]. These findings underline the importance of considering both age and lipid profile as potential modifiers when evaluating dietary interventions for psychological states. Additional investigations are essential to support these conclusions and explore the underlying biological pathways.

This study is, to our knowledge, the largest community-based cohort study conducted in China that explores the relationship between adherence to the Mediterranean diet and incident depression. Notably, the prospective cohort design can support causal inferences, as dietary assessments were conducted prior to the onset of depression, thereby minimizing the risk of reverse causality commonly associated with cross-sectional studies. Unlike most previous research that relied on depressive symptoms that were assessed by scales or self-reported, we used clinically diagnosed cases of depression. Additionally, the comprehensive adjustment for potential confounders and the design of subgroup and sensitivity analyses enhanced the stability of our observations. Furthermore, the integration of individual-level data with local health information systems helped reduce the possibility of loss to follow-up. However, this study does have some limitations. Early-stage depression may lead to changes in dietary habits, potentially introducing reverse causality. To address this, we conducted lag-time analyses, which indicated that the correlation between aMED adherence and depression remained stable, even after excluding the initial one and two years of follow-up. Another limitation is that dietary intake was evaluated using self-reported questionnaires at baseline, and participants’ eating habits might have altered during the follow-up period, leading to the potential for exposure misclassification. Nevertheless, previous research suggests that dietary habits remain relatively consistent over time, making substantial changes less likely [66,67]. In addition, to reduce such bias, a validated FFQ was used in this study, with correlation coefficients ranging from 0.20 to 0.60 [68]. Finally, since this study was observational, the possibility of residual confounding from variables that were not measured or accounted for, including stress-related influences, cannot be entirely discounted.

## 5. Conclusions

Higher adherence to the Mediterranean diet was associated with a lower risk of incident depression in this Chinese population. Owing to the insufficient consumption of vegetables, fruits, and whole grains, the Mediterranean diet is not a typical dietary pattern among the Chinese population, despite being widely practiced in Mediterranean countries. Consequently, adopting the Mediterranean diet may provide greater health benefits in the Chinese population, particularly in the context of the increasing focus on healthier lifestyles, including dietary improvements [69].

## Figures and Tables

**Table 1:** Baseline characteristics of participants according to their adherence to aMED.

Characteristics		Adherence to the aMED, N (%)			*p* Value
	Overall (*n* = 52,232)	Low (0–3) (*n* = 18,294)	Moderate (4–5) (*n* = 22,960)	High (6–9) (*n* = 10,978)	
**Age (years)**	58 (50–65)	59 (51–65)	57 (49–64)	58 (50–65)	<0.001
**Males**	20,201 (38.7)	6753 (36.9)	9051 (39.4)	4397 (40.1)	<0.001
**Educational attainment**					<0.001
Junior high school or below	37,232 (71.3)	14,627 (80.0)	15,969 (69.5)	6636 (60.5)	
High school or above	15,000 (28.7)	3667 (20.0)	6991 (30.5)	4342 (39.5)	
**Marital status**					<0.001
Married	47,688 (91.3)	16,750 (91.6)	20,968 (91.3)	9970 (90.8)	
Unmarried	1082 (2.1)	315 (1.7)	530 (2.3)	237 (2.2)	
Divorced or other	3462 (6.6)	1229 (6.7)	1462 (6.4)	771 (7.0)	
**Retired**	31,400 (60.1)	11,272 (61.6)	13,294 (57.9)	6834 (62.3)	<0.001
**Total energy intake (kcal/d)**	1298.5 (1015.1–1671.0)	1078.3 (872.1–1375.4)	1345.3 (1073.7–1700.0)	1583.2 (1285.7–1973.3)	<0.001
**BMI level**					0.041
Normal	25,746 (49.3)	9031 (49.4)	11,440 (49.8)	5275 (48.1)	
Overweight	20,033 (38.4)	7026 (38.4)	8688 (37.8)	4319 (39.3)	
Obese	6453 (12.3)	2237 (12.2)	2832 (12.4)	1384 (12.6)	
**Smoking status**					<0.001
Never	40,695 (77.9)	14,400 (78.7)	17,880 (77.9)	8415 (76.6)	
Stopped ≥ 10 years	633 (1.2)	243 (1.3)	273 (1.2)	117 (1.1)	
Stopped < 10 years	502 (1.0)	178 (1.0)	215 (0.9)	109 (1.0)	
Current	10,402 (19.9)	3473 (19.0)	4592 (20.0)	2337 (21.3)	
**PSQI**	4 (2–6)	4 (2–6)	4 (2–6)	4 (2–6)	<0.001 ^1^
**Physical activity level**					0.214
Q1	23,353 (44.7)	8101 (44.3)	10,334 (45.0)	4918 (44.8)	
Q2	11,832 (22.7)	4134 (22.6)	5251 (22.9)	2447 (22.3)	
Q3	17,047 (32.6)	6059 (33.1)	7375 (32.1)	3613 (32.9)	
**Family history of depression**	291 (0.6)	98 (0.5)	132 (0.6)	61 (0.6)	0.868
**History of chronic diseases**					
Hypertension	24,988 (47.8)	8773 (48.0)	10,982 (47.8)	5233 (47.7)	0.892
Diabetes	7085 (13.6)	2525 (13.8)	3100 (13.5)	1460 (13.3)	0.445
Hyperlipidemia	29,415 (56.3)	10,244 (56.0)	12,833 (55.9)	6338 (57.7)	0.003
Dementia	111 (0.2)	44 (0.2)	41 (0.2)	26 (0.2)	0.328
Parkinson’s disease	188 (0.4)	68 (0.4)	83 (0.4)	37 (0.3)	0.890

^1^ Although the same median (4) and interquartile range (2–6) were observed across all groups, the Kruskal–Wallis test revealed a significant difference in PSQI scores (*p* < 0.001). This apparent contradiction can be explained by the nature of the Kruskal–Wallis test, which is sensitive to variations in the distribution shape and rank order, rather than simply comparing central tendency measures. aMED: alternate Mediterranean diet; BMI: body mass index; PSQI: Pittsburgh Sleep Quality Index; Q: quintile.

**Table 2:** Associations between adherence to the aMED and risk of incident depression.

Adherence to the aMED	Cases/Person-Year	HR (95% CI)		
		Model 1 ^1^	Model 2 ^2^	Model 3 ^3^
Low (0–3)	517/119,729	Reference	Reference	Reference
Moderate (4–5)	495/144,031	0.82 (0.73, 0.93)	0.87 (0.77, 0.99)	0.87 (0.76, 0.99)
High (6–9)	208/64,592	0.74 (0.63, 0.87)	0.83 (0.70, 0.99)	0.83 (0.70, 0.98)
Per unit increase		0.92 (0.89, 0.96)	0.95 (0.91, 0.99)	0.95 (0.91, 0.99)
*p*-trend		<0.001	0.010	0.009

^1^ Cox proportional hazard regression model adjusted for age and gender. ^2^ Cox proportional hazard regression model further adjusted for educational attainment, BMI, marital status, retirement status, total energy intake, physical activity, PSQI, smoking status, and family history of depression. ^3^ Cox proportional hazard regression model further adjusted for history of hypertension, diabetes, hyperlipidemia, dementia, and Parkinson’s disease. *p*-trends were obtained by analyzing the aMED score as a continuous variable in the models. HR: hazard ratio; 95% CI: 95% confidence interval; aMED: alternate Mediterranean diet.

## Data Availability

The dataset used in this study is available from the corresponding author upon reasonable request.

## Supplementary Materials

The following supporting information can be downloaded at: https://www.mdpi.com/article/10.3390/nu17060942/s1, Table S1: Dietary intake of aMED components across different levels of adherence; Table S2: Associations between adherence to the aMED and risk of depression after exclusion of participants diagnosed with depression within the initial 1 and 2 years of follow-up; Table S3: Associations between the aMED group and risk of depression after removing alcohol from the aMED components; Table S4: Associations between the level of aMED score and risk of depression.

## Abbreviations

aMED	Alternate Mediterranean diet score
MD	Mediterranean diet
SSACB	Shanghai Suburban Adult Cohort and Biobank
FFQ	Food frequency questionnaire
ICD-10	International Classification of Diseases, Tenth Revision
BMI	Body mass index
CHD	Coronary heart disease
EMRS	Electronic medical record system
IPAQ	International physical activity questionnaire
MET	Metabolic equivalent of task
PSQI	Pittsburgh Sleep Quality Index
RCS	Restricted cubic spline
BDNF	Brain-derived neurotrophic factor
HDL	High-density lipoprotein

## References

[1] GBD 2019 Mental Disorders Collaborators (2022). Global, Regional, and National Burden of 12 Mental Disorders in 204 Countries and Territories, 1990–2019: A Systematic Analysis for the Global Burden of Disease Study 2019. Lancet Psychiatry.

[2] Nichols E., Steinmetz J.D., Vollset S.E., Fukutaki K., Chalek J., Abd-Allah F., Abdoli A., Abualhasan A., Abu-Gharbieh E., Akram T.T. (2022). Estimation of the Global Prevalence of Dementia in 2019 and Forecasted Prevalence in 2050: An Analysis for the Global Burden of Disease Study 2019. Lancet Public Health.

[3] Lu J., Xu X., Huang Y., Li T., Ma C., Xu G., Yin H., Xu X., Ma Y., Wang L. (2021). Prevalence of Depressive Disorders and Treatment in China: A Cross-Sectional Epidemiological Study. Lancet Psychiatry.

[4] Buckman J.E., Underwood A., Clarke K., Saunders R., Hollon S.D., Fearon P., Pilling S. (2018). Risk Factors for Relapse and Recurrence of Depression in Adults and How They Operate: A Four-Phase Systematic Review and Meta-Synthesis. Clin. Psychol. Rev..

[5] Hammen C. (2018). Risk Factors for Depression: An Autobiographical Review. Annu. Rev. Clin. Psychol..

[6] Zhang M.-M., Ma Y., Du L.-T., Wang K., Li Z., Zhu W., Sun Y.-H., Lu L., Bao Y.-P., Li S.-X. (2022). Sleep Disorders and Non-Sleep Circadian Disorders Predict Depression: A Systematic Review and Meta-Analysis of Longitudinal Studies. Neurosci. Biobehav. Rev..

[7] Pearce M., Garcia L., Abbas A., Strain T., Schuch F.B., Golubic R., Kelly P., Khan S., Utukuri M., Laird Y. (2022). Association Between Physical Activity and Risk of Depression: A Systematic Review and Meta-Analysis. JAMA Psychiatry.

[8] Marx W., Lane M., Hockey M., Aslam H., Berk M., Walder K., Borsini A., Firth J., Pariante C.M., Berding K. (2021). Diet and Depression: Exploring the Biological Mechanisms of Action. Mol. Psychiatry.

[9] Zajkowska Z., Walsh A., Zonca V., Gullett N., Pedersen G.A., Kieling C., Swartz J.R., Karmacharya R., Fisher H.L., Kohrt B.A. (2021). A Systematic Review of the Association between Biological Markers and Environmental Stress Risk Factors for Adolescent Depression. J. Psychiatr. Res..

[10] Brandt L., Liu S., Heim C., Heinz A. (2022). The Effects of Social Isolation Stress and Discrimination on Mental Health. Transl. Psychiatry.

[11] Sarris J., Logan A.C., Akbaraly T.N., Amminger G.P., Balanzá-Martínez V., Freeman M.P., Hibbeln J., Matsuoka Y., Mischoulon D., Mizoue T. (2015). Nutritional Medicine as Mainstream in Psychiatry. Lancet Psychiatry.

[12] Yin W., Löf M., Chen R., Hultman C.M., Fang F., Sandin S. (2021). Mediterranean Diet and Depression: A Population-Based Cohort Study. Int. J. Behav. Nutr. Phys. Act..

[13] Matison A.P., Mather K.A., Flood V.M., Reppermund S. (2021). Associations between Nutrition and the Incidence of Depression in Middle-Aged and Older Adults: A Systematic Review and Meta-Analysis of Prospective Observational Population-Based Studies. Ageing Res. Rev..

[14] Matsuoka Y.J., Sawada N., Mimura M., Shikimoto R., Nozaki S., Hamazaki K., Uchitomi Y., Tsugane S. (2017). Dietary Fish, n-3 Polyunsaturated Fatty Acid Consumption, and Depression Risk in Japan: A Population-Based Prospective Cohort Study. Transl. Psychiatry.

[15] Wang P., Song M., Eliassen A.H., Wang M., Fung T.T., Clinton S.K., Rimm E.B., Hu F.B., Willett W.C., Tabung F.K. (2023). Optimal Dietary Patterns for Prevention of Chronic Disease. Nat. Med..

[16] Metcalfe-Roach A., Yu A.C., Golz E., Cirstea M., Sundvick K., Kliger D., Foulger L.H., Mackenzie M., Finlay B.B., Appel-Cresswell S. (2021). MIND and Mediterranean Diets Associated with Later Onset of Parkinson’s Disease. Mov. Disord..

[17] Ballarini T., Melo van Lent D., Brunner J., Schröder A., Wolfsgruber S., Altenstein S., Brosseron F., Buerger K., Dechent P., Dobisch L. (2021). Mediterranean Diet, Alzheimer Disease Biomarkers and Brain Atrophy in Old Age. Neurology.

[18] Coelho-Júnior H.J., Trichopoulou A., Panza F. (2021). Cross-Sectional and Longitudinal Associations between Adherence to Mediterranean Diet with Physical Performance and Cognitive Function in Older Adults: A Systematic Review and Meta-Analysis. Ageing Res. Rev..

[19] Trichopoulou A., Costacou T., Bamia C., Trichopoulos D. (2003). Adherence to a Mediterranean Diet and Survival in a Greek Population. N. Engl. J. Med..

[20] Fung T.T., Rexrode K.M., Mantzoros C.S., Manson J.E., Willett W.C., Hu F.B. (2009). Mediterranean Diet and Incidence of and Mortality from Coronary Heart Disease and Stroke in Women. Circulation.

[21] Crous-Bou M., Fung T.T., Prescott J., Julin B., Du M., Sun Q., Rexrode K.M., Hu F.B., De Vivo I. (2014). Mediterranean Diet and Telomere Length in Nurses’ Health Study: Population Based Cohort Study. BMJ.

[22] Makarem N., Chau K., Miller E.C., Gyamfi-Bannerman C., Tous I., Booker W., Catov J.M., Haas D.M., Grobman W.A., Levine L.D. (2022). Association of a Mediterranean Diet Pattern with Adverse Pregnancy Outcomes Among US Women. JAMA Netw. Open.

[23] Chen E.Y., Mahurkar-Joshi S., Liu C., Jaffe N., Labus J.S., Dong T.S., Gupta A., Patel S., Mayer E.A., Chang L. (2024). The Association Between a Mediterranean Diet and Symptoms of Irritable Bowel Syndrome. Clin. Gastroenterol. Hepatol..

[24] De Vries E., Van Schrojenstein Lantman M., Hoebregts V., Mackus M., Garssen J., Verster J.C., Scholey A. (2017). Mediterranean Diet and Mood. Eur. Neuropsychopharmacol..

[25] Esgunoglu L., Jennings A., Connole E.S., Murphy K.J., Minihane A.M. (2022). Short-Term Effects of a Mediterranean-Style Dietary Pattern on Cognition and Mental Well-Being: A Systematic Review of Clinical Trials. Br. J. Nutr..

[26] Shafiei F., Salari-Moghaddam A., Larijani B., Esmaillzadeh A. (2019). Adherence to the Mediterranean Diet and Risk of Depression: A Systematic Review and Updated Meta-Analysis of Observational Studies. Nutr. Rev..

[27] Lassale C., Batty G.D., Baghdadli A., Jacka F., Sánchez-Villegas A., Kivimäki M., Akbaraly T. (2019). Healthy Dietary Indices and Risk of Depressive Outcomes: A Systematic Review and Meta-Analysis of Observational Studies. Mol. Psychiatry.

[28] Gianfredi V., Koster A., Odone A., Amerio A., Signorelli C., Schaper N.C., Bosma H., Köhler S., Dagnelie P.C., Stehouwer C.D.A. (2021). Associations of Dietary Patterns with Incident Depression: The Maastricht Study. Nutrients.

[29] Hershey M.S., Sanchez-Villegas A., Sotos-Prieto M., Fernandez-Montero A., Pano O., Lahortiga-Ramos F., Martínez-González M.Á., Ruiz-Canela M. (2022). The Mediterranean Lifestyle and the Risk of Depression in Middle-Aged Adults. J. Nutr..

[30] Marozoff S., Veugelers P.J., Dabravolskaj J., Eurich D.T., Ye M., Maximova K. (2020). Diet Quality and Health Service Utilization for Depression: A Prospective Investigation of Adults in Alberta’s Tomorrow Project. Nutrients.

[31] Lugon G., Hernáez Á., Jacka F.N., Marrugat J., Ramos R., Garre-Olmo J., Elosua R., Lassale C. (2024). Association between Different Diet Quality Scores and Depression Risk: The REGICOR Population-Based Cohort Study. Eur. J. Nutr..

[32] Sun J., Li Z., Li Y., Zhang D. (2021). Intakes of Specific Categories of Vegetables and Fruits Are Inversely Associated with Depressive Symptoms Among Adults. J. Epidemiol..

[33] Yang R., Wang L., Jin K., Cao S., Wu C., Guo J., Chen J., Tang H., Tang M. (2022). Omega-3 Polyunsaturated Fatty Acids Supplementation Alleviate Anxiety Rather Than Depressive Symptoms Among First-Diagnosed, Drug-Naïve Major Depressive Disorder Patients: A Randomized Clinical Trial. Front. Nutr..

[34] Su Q., Yu B., He H., Zhang Q., Meng G., Wu H., Du H., Liu L., Shi H., Xia Y. (2016). Nut Consumption Is Associated with Depressive Symptoms among Chinese Adults. Depress. Anxiety.

[35] Zhao Q., Chen B., Wang R., Zhu M., Shao Y., Wang N., Liu X., Zhang T., Jiang F., Wang W. (2020). Cohort Profile: Protocol and Baseline Survey for the Shanghai Suburban Adult Cohort and Biobank (SSACB) Study. BMJ Open.

[36] Yi K., Cui S., Tang M., Wu Y., Xiang Y., Yu Y., Tong X., Jiang Y., Zhao Q., Zhao G. (2022). Adherence to DASH Dietary Pattern and Its Association with Incident Hyperuricemia Risk: A Prospective Study in Chinese Community Residents. Nutrients.

[37] Martínez-González M.A., García-López M., Bes-Rastrollo M., Toledo E., Martínez-Lapiscina E.H., Delgado-Rodriguez M., Vazquez Z., Benito S., Beunza J.J. (2011). Mediterranean Diet and the Incidence of Cardiovascular Disease: A Spanish Cohort. Nutr. Metab. Cardiovasc. Dis..

[38] Mitrou P.N., Kipnis V., Thiébaut A.C.M., Reedy J., Subar A.F., Wirfält E., Flood A., Mouw T., Hollenbeck A.R., Leitzmann M.F. (2007). Mediterranean Dietary Pattern and Prediction of All-Cause Mortality in a US Population: Results from the NIH-AARP Diet and Health Study. Arch. Intern. Med..

[39] Wu H., Gu Y., Meng G., Wu H., Zhang S., Wang X., Zhang J., Huang T., Niu K. (2023). Quality of Plant-Based Diet and the Risk of Dementia and Depression among Middle-Aged and Older Population. Age Ageing.

[40] Craig C.L., Marshall A.L., Sjöström M., Bauman A.E., Booth M.L., Ainsworth B.E., Pratt M., Ekelund U., Yngve A., Sallis J.F. (2003). International Physical Activity Questionnaire: 12-Country Reliability and Validity. Med. Sci. Sports Exerc..

[41] Buysse D.J., Reynolds C.F., Monk T.H., Berman S.R., Kupfer D.J. (1989). The Pittsburgh Sleep Quality Index: A New Instrument for Psychiatric Practice and Research. Psychiatry Res..

[42] Anderson B.O., Berdzuli N., Ilbawi A., Kestel D., Kluge H.P., Krech R., Mikkelsen B., Neufeld M., Poznyak V., Rekve D. (2023). Health and Cancer Risks Associated with Low Levels of Alcohol Consumption. Lancet Public. Health.

[43] Li J., Wang H., Li M., Shen Q., Li X., Zhang Y., Peng J., Rong X., Peng Y. (2020). Effect of Alcohol Use Disorders and Alcohol Intake on the Risk of Subsequent Depressive Symptoms: A Systematic Review and Meta-Analysis of Cohort Studies. Addiction.

[44] Masana M.F., Haro J.M., Mariolis A., Piscopo S., Valacchi G., Bountziouka V., Anastasiou F., Zeimbekis A., Tyrovola D., Gotsis E. (2018). Mediterranean Diet and Depression among Older Individuals: The Multinational MEDIS Study. Exp. Gerontol..

[45] Hwang Y.-G., Pae C., Lee S.-H., Yook K.-H., Park C.I. (2023). Relationship between Mediterranean Diet and Depression in South Korea: The Korea National Health and Nutrition Examination Survey. Front. Nutr..

[46] Bizzozero-Peroni B., Martínez-Vizcaíno V., Fernández-Rodríguez R., Jiménez-López E., Núñez de Arenas-Arroyo S., Saz-Lara A., Díaz-Goñi V., Mesas A.E. (2025). The Impact of the Mediterranean Diet on Alleviating Depressive Symptoms in Adults: A Systematic Review and Meta-Analysis of Randomized Controlled Trials. Nutr. Rev..

[47] Saura-Calixto F., Goñi I. (2009). Definition of the Mediterranean Diet Based on Bioactive Compounds. Crit. Rev. Food Sci. Nutr..

[48] Corella D., Coltell O., Macian F., Ordovás J.M. (2018). Advances in Understanding the Molecular Basis of the Mediterranean Diet Effect. Annu. Rev. Food Sci. Technol..

[49] Bizzozero-Peroni B., Ortolá R., Martínez-Vizcaíno V., Rodríguez-Artalejo F., Fernández-Rodríguez R., Banegas J.R., Lopez-Garcia E., Mesas A.E. (2022). Proinflammatory Dietary Pattern and Depression Risk in Older Adults: Prospective Analyses from the Seniors-ENRICA Studies. Clin. Nutr..

[50] Cox I.J., Idilman R., Fagan A., Turan D., Ajayi L., Le Guennec A.D., Taylor-Robinson S.D., Karakaya F., Gavis E., Andrew Atkinson R. (2020). Metabolomics and Microbial Composition Increase Insight into the Impact of Dietary Differences in Cirrhosis. Liver Int..

[51] Maskarinec G., Hullar M.A.J., Monroe K.R., Shepherd J.A., Hunt J., Randolph T.W., Wilkens L.R., Boushey C.J., Le Marchand L., Lim U. (2019). Fecal Microbial Diversity and Structure Are Associated with Diet Quality in the Multiethnic Cohort Adiposity Phenotype Study. J. Nutr..

[52] Meslier V., Laiola M., Roager H.M., De Filippis F., Roume H., Quinquis B., Giacco R., Mennella I., Ferracane R., Pons N. (2020). Mediterranean Diet Intervention in Overweight and Obese Subjects Lowers Plasma Cholesterol and Causes Changes in the Gut Microbiome and Metabolome Independently of Energy Intake. Gut.

[53] Guo S., Kim W.J., Lok J., Lee S.-R., Besancon E., Luo B.-H., Stins M.F., Wang X., Dedhar S., Lo E.H. (2008). Neuroprotection via Matrix-Trophic Coupling between Cerebral Endothelial Cells and Neurons. Proc. Natl. Acad. Sci. USA.

[54] Sánchez-Villegas A., Galbete C., Martinez-González M.A., Martinez J.A., Razquin C., Salas-Salvadó J., Estruch R., Buil-Cosiales P., Martí A. (2011). The Effect of the Mediterranean Diet on Plasma Brain-Derived Neurotrophic Factor (BDNF) Levels: The PREDIMED-NAVARRA Randomized Trial. Nutr. Neurosci..

[55] Sanllorente A., Soria-Florido M.T., Castañer O., Lassale C., Salas-Salvadó J., Martínez-González M.Á., Subirana I., Ros E., Corella D., Estruch R. (2021). A Lifestyle Intervention with an Energy-Restricted Mediterranean Diet and Physical Activity Enhances HDL Function: A Substudy of the PREDIMED-Plus Randomized Controlled Trial. Am. J. Clin. Nutr..

[56] Chourpiliadis C., Zeng Y., Lovik A., Wei D., Valdimarsdóttir U., Song H., Hammar N., Fang F. (2024). Metabolic Profile and Long-Term Risk of Depression, Anxiety, and Stress-Related Disorders. JAMA Netw. Open.

[57] Huang Y., Wang Y.U., Wang H., Liu Z., Yu X., Yan J., Yu Y., Kou C., Xu X., Lu J. (2019). Prevalence of Mental Disorders in China: A Cross-Sectional Epidemiological Study. Lancet Psychiatry.

[58] Baierle M., Nascimento S.N., Moro A.M., Brucker N., Freitas F., Gauer B., Durgante J., Bordignon S., Zibetti M., Trentini C.M. (2015). Relationship between Inflammation and Oxidative Stress and Cognitive Decline in the Institutionalized Elderly. Oxidative Med. Cell. Longev..

[59] Rawdin B.J., Mellon S.H., Dhabhar F.S., Epel E.S., Puterman E., Su Y., Burke H.M., Reus V.I., Rosser R., Hamilton S.P. (2013). Dysregulated Relationship of Inflammation and Oxidative Stress in Major Depression. Brain Behav. Immun..

[60] Bhatt S., Nagappa A.N., Patil C.R. (2020). Role of Oxidative Stress in Depression. Drug Discov. Today.

[61] Pitsavos C., Panagiotakos D.B., Tzima N., Chrysohoou C., Economou M., Zampelas A., Stefanadis C. (2005). Adherence to the Mediterranean Diet Is Associated with Total Antioxidant Capacity in Healthy Adults: The ATTICA Study2. Am. J. Clin. Nutr..

[62] Estruch R. (2010). Anti-Inflammatory Effects of the Mediterranean Diet: The Experience of the PREDIMED Study. Proc. Nutr. Soc..

[63] Jimenez-Torres J., Alcalá-Diaz J.F., Torres-Peña J.D., Gutierrez-Mariscal F.M., Leon-Acuña A., Gómez-Luna P., Fernández-Gandara C., Quintana-Navarro G.M., Fernandez-Garcia J.C., Perez-Martinez P. (2021). Mediterranean Diet Reduces Atherosclerosis Progression in Coronary Heart Disease: An Analysis of the CORDIOPREV Randomized Controlled Trial. Stroke.

[64] Hong N., Lin Y., Ye Z., Yang C., Huang Y., Duan Q., Xie S. (2022). The Relationship between Dyslipidemia and Inflammation among Adults in East Coast China: A Cross-Sectional Study. Front. Immunol..

[65] Esteve E., Ricart W., Fernández-Real J.M. (2005). Dyslipidemia and Inflammation: An Evolutionary Conserved Mechanism. Clin. Nutr..

[66] Appel L.J., Brands M.W., Daniels S.R., Karanja N., Elmer P.J., Sacks F.M., American Heart Association (2006). Dietary Approaches to Prevent and Treat Hypertension: A Scientific Statement from the American Heart Association. Hypertension.

[67] Goode A.D., Reeves M.M., Eakin E.G. (2012). Telephone-Delivered Interventions for Physical Activity and Dietary Behavior Change: An Updated Systematic Review. Am. J. Prev. Med..

[68] Cui Q., Xia Y., Wu Q., Chang Q., Niu K., Zhao Y. (2023). Validity of the Food Frequency Questionnaire for Adults in Nutritional Epidemiological Studies: A Systematic Review and Meta-Analysis. Crit. Rev. Food Sci. Nutr..

[69] Li B., Ma X., Yu Y., Wang G., Zhuang N., Liu H., Wu H., Zhang H., Yu F., Hou Y., Li B. (2020). “Healthy China 2030”: Promoting Health and Longevity of the Whole Nation. Tutorial for Outline of the Healthy China 2030 Plan.

