# Supplementary material for: Adherence to the Mediterranean Diet and Risk of Depression: A Cohort Study in Chinese Community Residents
Source: Nutrients. 2025 Mar 7;17(6):942. doi: 10.3390/nu17060942 (PMC11945095; doi:10.3390/nu17060942)
Supplement: Supplementary file 1 [file nutrients-17-00942-s001.zip › nutrients-3493110-supplementary.pdf]

Table S1. Dietary intake of aMED components across different levels of adherence

Characteristics	Overall (n=52,232)	Adherence to the aMED, median [IQR] Low (0-3) (n=18,294)	Moderate (4-5) (n=22,960)	High (6-9) (n=10,978)	*P* value
Whole grains (g/week)	46.0 [8.3, 200.0]	23.0 [0.0, 46.0]	69.0 [11.6, 200.0]	200.0 [69.0, 300.0]	<0.001
Vegetables (excluding potatoes) (g/week)	1400.0 [700.0, 2100.0]	700.0 [350.0, 1400.0]	1400.0 [700.0, 2100.0]	1750.0 [1400.0, 2800.0]	<0.001
Fruits (g/week)	700.0 [200.0, 1050.0]	300.0 [100.0, 700.0]	700.0 [300.0, 1050.0]	1050.0 [700.0, 1400.0]	<0.001
Nuts (g/week)	36.8 [5.8, 100.0]	9.2 [0.0, 23.0]	46.0 [9.2, 100.0]	100.0 [55.2, 250.0]	<0.001
Legumes (serving/week)	2.3 [0.8, 4.2]	1.5 [0.4, 2.0]	3.3 [1.2, 4.6]	3.9 [3.3, 6.7]	<0.001
The ratio of monounsaturated to saturated fats	1.3 [1.2, 1.5]	1.3 [1.1, 1.5]	1.3 [1.2, 1.5]	1.4 [1.3, 1.5]	<0.001
Alcohol (g/week)	0.0 [0.0, 0.0]	0.0 [0.0, 0.0]	0.0 [0.0, 0.0]	0.0 [0.0, 0.0]	<0.001
Red and processed meats (g/week)	257.5 [146.0, 450.0]	255.8 [123.0, 446.0]	270.0 [161.2, 490.0]	246.0 [151.8, 442.1]	<0.001
Fish (g/week)	360.0 [200.0, 600.0]	223.0 [123.0, 317.3]	400.0 [243.6, 600.0]	600.0 [411.5, 800.0]	<0.001

aMED: the alternate Mediterranean diet; IQR: interquartile range

Table S2. Associations between adherence to the aMED and risk of depression after exclusion of participants diagnosed with depression within the initial 1 and 2 years of follow-up

Adherence to the aMED	HR (95%CI)		
	**Model 1[a]**	**Model 2[b]**	**Model 3[c]**
**Excluding patients within the 1 year**			
Low (score 0-3)	Reference	Reference	Reference
Moderate (score 4-5)	**0.85 (0.74, 0.98)**	0.90 (0.77, 1.04)	0.89 (0.77, 1.03)
High (score 6-9)	**0.72 (0.60, 0.87)**	**0.81 (0.66, 0.99)**	**0.81 (0.66, 0.98)**
Per unit increase	**0.92 (0.88, 0.96)**	**0.94 (0.90, 0.98)**	**0.94 (0.90, 0.98)**
P-trend	< 0.001	0.011	0.010
**Excluding patients within the 2 years**			
Low (score 0-3)	Reference	Reference	Reference
Moderate (score 4-5)	0.88 (0.75, 1.02)	0.94 (0.79, 1.10)	0.93 (0.79, 1.10)
High (score 6-9)	**0.65 (0.53, 0.82)**	**0.75 (0.59, 0.95)**	**0.75 (0.59, 0.95)**
Per unit increase	**0.91 (0.87, 0.95)**	**0.93 (0.89, 0.98)**	**0.93 (0.89, 0.98)**
P-trend	< 0.001	0.009	0.008

HR: hazard ratio; 95% CI: 95% confidence interval; aMED: the alternate Mediterranean diet;

[a] Cox proportional hazard regression model adjusted for age and gender.

[b] Cox proportional hazard regression model further adjusted for educational attainment, BMI, marital status, retirement status, total energy intake, physical activity, PSQI, smoking status, and family history of depression.

[c] Cox proportional hazard regression model further adjusted for history of hypertension, diabetes, hyperlipidemia, dementia, and Parkinson's disease.

P-trends were obtained by analyzing the aMED score as a continuous variable in the models. Data with *P* values below 0.05 are presented in bold type.

Table S3. Associations between the aMED group and risk of depression after removing alcohol from the aMED components

Adherence to the aMED	HR (95%CI)		
	**Model 1[a]**	**Model 2[b]**	**Model 3[c]**
Score 0-2	Reference	Reference	Reference
Score 3-5	0.76 (0.66, 0.87)	0.81 (0.70, 0.93)	0.80 (0.70, 0.93)
Score 6-8	0.67 (0.56, 0.80)	0.76 (0.63, 0.93)	0.76 (0.62, 0.93)
Per unit increase	0.92 (0.89, 0.95)	0.95 (0.91, 0.99)	0.95 (0.91, 0.98)
P-trend	< 0.001	0.007	0.007

[a] Cox proportional hazard regression model adjusted for age and gender.

[b] Cox proportional hazard regression model further adjusted for educational attainment, BMI, marital status, retirement status, total energy intake, physical activity, PSQI, smoking status, family history of depression and drinking status.

[c] Cox proportional hazard regression model further adjusted for history of hypertension, diabetes, hyperlipidemia, dementia, and Parkinson's disease.

P-trends were obtained by analyzing the aMED score as a continuous variable in the models.

Table S4. Associations between the level of aMED score and risk of depression

AMED score level	Cases/person-year	HR (95%CI)		
		Model 1[a]	Model 2[b]	Model 3[c]
Q1 (score 0-3)	517/119729	Reference	Reference	Reference
Q2 (score 4)	262/76285	**0.826 (0.712, 0.959)**	**0.857 (0.737, 0.997)**	**0.855 (0.735, 0.995)**
Q3 (score 5)	233/67746	**0.822 (0.704, 0.959)**	0.890 (0.757, 1.046)	0.888 (0.755, 1.043)
Q4 (score 6-9)	208/64592	**0.739 (0.629, 0.868)**	**0.833 (0.670, 0.992)**	**0.830 (0.697, 0.988)**

[a] Cox proportional hazard regression model adjusted for age and gender.

[b] Cox proportional hazard regression model further adjusted for educational attainment, BMI, marital status, retirement status, total energy intake, physical activity, PSQI, smoking status, and family history of depression.

[c] Cox proportional hazard regression model further adjusted for history of hypertension, diabetes, hyperlipidemia, dementia, and Parkinson's disease.

Data with *P* values below 0.05 are presented in bold type.
